# The unified cardiometabolic disease continuum: mechanistic stages of a single pathophysiological process

**DOI:** 10.3389/fendo.2026.1897556

**Published:** 2026-07-09

**Authors:** Luz María Quirino-Vela, Miguel A. Mayoral-Chávez, Carlos A. Matías-Cervantes, Yobana Pérez-Cervera, Iván A. García-Montalvo, Juan Alpuche

**Affiliations:** 1 Laboratorio de Bioquímica, Facultad de Medicina y Cirugía, Universidad Autónoma Benito Juárez de Oaxaca (UABJO), Oaxaca de Juárez, Oaxaca, Mexico; 2 SECIHTI-Facultad de Medicina y Cirugía, UABJO, Oaxaca de Juárez, Oaxaca, Mexico; 3 Centro de Estudios en Ciencias de la Salud y la Enfermedad, Facultad de Odontología, Universidad Autónoma Benito Juárez de Oaxaca, Oaxaca de Juárez, Oaxaca, Mexico; 4 División de Estudios de Posgrado e Investigación, Tecnológico Nacional de México/Instituto Tecnológico de Oaxaca, Oaxaca, Mexico

**Keywords:** cardiometabolic continuum, cardiometabolic risk, ceramides, epicardial adipose tissue, gut microbiome, NLRP3 inflammasome, pathophysiological processes, unified cardiometabolic disease

## Abstract

**Background:**

Type 2 diabetes mellitus (T2DM), atherosclerotic cardiovascular disease (ASCVD), heart failure with preserved ejection fraction (HFpEF), metabolic dysfunction-associated steatotic liver disease (MASLD), hypertension, and chronic kidney disease (CKD) share risk factors and may represent endpoints of a pathophysiological cardiometabolic continuum. We analyzed data suggesting these phenotypes arise along a unified cardiometabolic disease (UCD) continuum with distinct stages, biomarkers, and therapeutic targets.

**Methods:**

A structured narrative review was conducted using PubMed/MEDLINE, Scopus, Web of Science, and Google Scholar. Searches combined terms for mechanistic drivers (visceral adipose tissue, ceramides, lipotoxicity, NF-κB/NLRP3 signaling, gut microbiota, TMAO, adipokines, endothelial dysfunction, epicardial fat, metabolic flexibility) and clinical endpoints. Priority was given to peer-reviewed translational studies, major outcome trials, and consensus statements published between 2017-2026.

**Results:**

Current evidence supports a mechanistic framework in which visceral adipose tissue dysfunction and ectopic lipid accumulation, progressing through ceramide-mediated lipotoxicity, NF-κB/NLRP3-driven inflammation, gut microbiome-derived endotoxemia and TMAO, adipokine dysregulation, endothelial dysfunction, epicardial fat-mediated cardiac remodeling, impaired metabolic flexibility, and a cardiorenal amplification loop. Stage-specific mediators (e.g., ceramides, NLRP3, TMAO, leptin-adiponectin ratio) serve as biomarkers. The benefits of GLP-1 receptor agonists, SGLT2 inhibitors, and finerenone across T2DM, ASCVD, HFpEF, MASLD, and CKD reflect pharmacologic modulation of this continuum.

**Discussion:**

Data support a UCD model where T2DM, ASCVD, MASLD, HFpEF, hypertension, and CKD are manifestations of a progressive pathophysiological continuum. Framing these conditions as stages of a continuum informs risk stratification, biomarker development, and mechanism-guided therapy, providing a framework for designing trials targeting the continuum rather than individual endpoints.

## 1 Introduction

Non-communicable diseases account for approximately 75% of global deaths, with cardiovascular disease (CVD) as the leading contributor, responsible for an estimated 19.2 million deaths in 2023, a rise from 13.1 million in 1990, equivalent to a 1.4-fold increase in cardiovascular disability-adjusted life years (DALYs) over the same period (1). Metabolic syndrome prevalence has more than doubled globally since 2000, rising from 14.7% to 31.0% in women and from 9.0% to 25.7% in men by 2023, affecting approximately 1.54 billion adults worldwide (2). Approximately 79.6% of CVD DALYs are attributable to modifiable cardiometabolic risk factors, dominated by high systolic blood pressure, dietary risks, elevated LDL cholesterol, high body mass index, and high fasting plasma glucose (1).

For decades, T2DM, ASCVD, HFpEF, MASLD, hypertension, and CKD have been managed as separate entities, each with its own specialist, guidelines, and therapeutic targets. This compartmentalized approach is increasingly impractical. Mechanistic studies, outcome trials, and longitudinal cohorts show these conditions may be interpreted as co-evolving expressions of shared pathological processes (3). The shift from a multi-disease model to a unified cardiometabolic disease (UCD) pathophysiological paradigm has profound implications. While clinical coding and guidelines still treat them as distinct diseases, at the mechanistic level, they may be usefully interpreted as overlapping, staged expressions of shared cardiometabolic biology, demanding therapies that target a shared core rather than isolated endpoints (4).

A growing body of evidence supports this shift from a multi−disease paradigm to a unified cardiometabolic disease (UCD) continuum. GLP−1 receptor agonists, originally developed for glycemic control, concurrently reduce MACE, HFpEF hospitalizations, and hepatic steatosis, consistent with modulation of a shared biological process expressed across multiple organs rather than independent “off−target” effects. Similarly, SGLT2 inhibitors reduce HFpEF, CKD progression, and ASCVD events in both diabetic and non-diabetic patients consistent with the presence of shared mechanistic roots. Clinically distinct entities such as MASLD and HFpEF now carry the descriptor “NASH of the heart,” reflecting a shared adiposity−driven lipotoxic and fibrotic substrate (5). The AHA cardiovascular–kidney–metabolic (CKM) framework is an important acknowledgment of this interconnectedness; however, it remains a clinical staging system based on organ comorbidity rather than a mechanistic disease (6). Therefore, this review proposes a mechanistic UCD continuum, in which each biological axis is interpreted here not only as a risk factor but also as a potential stage or amplifier in cardiometabolic progression, from VAT dysfunction to cardiorenal failure, highlighting mechanistic nodes amenable to biomarker development and therapeutic intervention.

## 2 Methods

A structured narrative review was conducted using PubMed/MEDLINE, Scopus, Web of Science, and Google Scholar. Searches combined Medical Subject Headings (MeSH) and free-text terms covering each proposed mechanistic stage of the unified cardiometabolic disease (UCD) continuum, including “visceral adipose tissue,” “ceramide,” “lipotoxicity,” “NLRP3 inflammasome,” “NF-κB,” “gut microbiota,” “TMAO,” “metabolic endotoxemia,” “epicardial adipose tissue,” “unified cardiometabolic disease,” and “cardiometabolic continuum.” These terms were combined with clinical endpoint terms including “type 2 diabetes,” “MASLD,” “HFpEF,” “ASCVD,” “CKD,” “hypertension,” and “atherosclerosis.” Priority was given to peer-reviewed reviews, meta-analyses, clinical guidelines, major cardiovascular or renal outcome trials, human cohort studies, translational studies, and seminal mechanistic articles published between January 2017 and May 2026. Foundational earlier references were also included when they were mechanistically essential to the proposed continuum. Articles with full-text availability were screened by two authors. Approximately 3,200 records were identified, approximately 980 were screened, 312 full texts were assessed, and 48 articles were included after applying the following inclusion criteria: peer-reviewed publication, mechanistic or clinical focus on cardiometabolic disease, relevance to at least one proposed UCD stage, and human or translational relevance.

### 2.1 Evidence appraisal and reliability classification

This review suggests a mechanistic pathophysiological framework instead of evaluating a single treatment. Therefore, evidence was not assessed using the formal therapeutic GRADE method. Instead, each mechanistic area was categorized based on the type of source, consistency of results, relevance to humans, translational strength, and level of clinical validation. Evidence was considered to have high reliability when supported by consistent human data, major clinical guidelines, large outcome trials, meta-analyses, or reproducible human cohort, imaging, or biomarker studies. Evidence was considered to have moderate reliability when supported by consistent human observational or translational evidence with strong biological plausibility but incomplete causal or interventional confirmation. Evidence was considered low or emerging when derived mainly from preclinical models, *in vitro* studies, early-phase trials, biomarker associations, or mechanistic plausibility without definitive validation in human outcome studies. This classification was used to distinguish high-confidence clinical mechanisms, such as visceral adiposity, endothelial dysfunction, albuminuria, cardiorenal risk amplification, and the cross-organ benefits of GLP-1 receptor agonists, SGLT2 inhibitors, finerenone, lifestyle intervention, RAAS inhibition, and lipid-lowering therapy, from more exploratory pathways, including selected inflammasome-, ceramide-, microbiome-, and senescence-targeted mechanisms. The resulting evidence appraisal is summarized in **Supplementary Table 1**.

## 3 Etiological drivers of the UCD continuum

### 3.1 Genetic and epigenetic determinants

The UCD continuum is not exclusively a result of VAT dysfunction; instead, VAT dysfunction constitutes the phenotypic expression of a complex upstream etiological framework. The polygenic risk architecture of obesity and T2DM includes variants in genes governing energy homeostasis (FTO, MC4R, PCSK1), adipogenesis (PPARG, ADRB3), insulin secretion (TCF7L2, KCNJ11, ABCC8), and lipid metabolism (APOE, LPL), whose cumulative burden determines susceptibility to VAT accumulation under conditions of positive energy balance (7). Beyond genomic sequence variation, epigenetic reprogramming plays an equally fundamental role: early-life exposures including intrauterine hyperglycemia, maternal obesity, and postnatal overnutrition induce persistent DNA methylation changes at promoters of key metabolic genes, priming adipose progenitor cells toward profibrotic and pro-inflammatory differentiation decades before clinical disease manifests (8).

Transgenerational epigenetic transmission of metabolic risk via paternal sperm small RNAs and maternal mitochondrial DNA heteroplasmy further amplifies susceptibility across generations independently of shared dietary environments (9). Histone acetylation patterns in white adipose progenitors, particularly at enhancers of TGF-β pathway genes, are durably altered by early caloric excess and resist normalization even after weight loss, a molecular basis for the clinical observation that bariatric intervention does not fully reverse cardiometabolic risk in individuals with long-standing obesity (8, 10). These genetic and epigenetic determinants define the individual’s “metabolic set point”, the threshold of cumulative energy surplus beyond which VAT expansion becomes pathological and the UCD continuum is initiated.

### 3.2 Central nervous system dysregulation and energy balance

The hypothalamic-pituitary-adrenal (HPA) axis occupies a central position in UCD pathogenesis as both a promoter and a consequence of VAT accumulation, constituting a bidirectional amplification loop. Chronic psychosocial stress, sleep deprivation, and circadian rhythm disruption activate the HPA axis, resulting in sustained hypercortisolemia (11). Cortisol acts via glucocorticoid receptors that are selectively enriched in omental adipocytes relative to subcutaneous fat depots: glucocorticoid receptor activation upregulates lipoprotein lipase and downregulates hormone-sensitive lipase in visceral fat, creating a biochemical milieu that preferentially directs circulating lipid toward VAT accumulation (12). Simultaneously, cortisol-driven hyperinsulinemia promotes further VAT expansion and directly suppresses adiponectin gene expression in omental adipocytes.

In addition to the HPA axis, hypothalamic leptin resistance diminishes the satiety signal that would typically limit energy intake, creating a state of functional hyperphagia despite adequate caloric stores. Impaired central GLP-1 signaling, further attenuates meal-induced satiety and incretin-mediated inhibition of hepatic glucose production (13). The net effect is a neuroendocrine environment that sustains positive energy balance independently of dietary composition, driving the chronic VAT expansion that initiates Stage I of the UCD continuum (10). Disruption of circadian rhythmicity desynchronizes peripheral metabolic clocks in liver, adipose, and pancreas from the central hypothalamic pacemaker, impairing insulin sensitivity by up to 30% and accelerating ectopic lipid deposition even in the absence of caloric excess. Together, these hypothalamic mechanisms constitute a central disturbance in appetite regulation, in which leptin resistance, impaired GLP-1 signaling, sleep disruption, chronic stress, and altered reward-pathway signaling reduce satiety, increase hedonic food intake, and sustain positive energy balance. This neuroendocrine dysregulation should therefore be interpreted as an upstream promoter of VAT expansion rather than merely a behavioral correlate of obesity.

### 3.3 Environmental, sedentary, and social determinants of health

Environmental and social determinants of health (SDOH) constitute indispensable upstream drivers of the UCD continuum, as recognized by the 2026 AHA/ACC/ADA/ASN Guideline on CKM Syndrome, “the increased likelihood of CKM syndrome and its adverse outcomes is further influenced by unfavorable conditions for lifestyle and self-care resulting from policies, economics, and the environment” (14). Food environment characteristics, density of ultra-processed food outlets, absence of grocery stores with fresh produce, and food insecurity, directly promote high-caloric, low-nutrient dietary patterns that sustain positive energy balance. Neighborhood-level walkability deficits and absence of safe spaces for physical activity reinforce sedentary behavior (15). Socioeconomic disadvantage elevates allostatic load through chronic stress, impairs access to preventive care, and concentrates obesogenic exposures in historically marginalized communities (16). High dietary sodium intake, frequently embedded within ultra-processed dietary patterns, represents an additional modifiable upstream promoter of the UCD continuum. Excess sodium intake contributes to plasma-volume expansion, endothelial dysfunction, arterial stiffness, RAAS/sympathetic activation, and obesity-related hypertension, thereby accelerating the transition from early metabolic dysfunction to Stage VI vascular injury and subsequent cardiorenal amplification (17).

Sedentary behavior operates as an independent UCD risk amplifier, physical inactivity impairs GLUT-4 expression and insulin-stimulated glucose uptake in skeletal muscle, reduces AMPK activation and attenuates mitochondrial biogenesis, worsening metabolic flexibility (Stage VIII of the UCD continuum) even before overt obesity develops (18). These pathways explain why sedentary individuals with a normal body mass index can exhibit Stage II–III UCD features (dyslipidemia, insulin resistance, early coronary atherosclerosis). The ADA Standards of Care 2026 emphasize that physical activity should be encouraged for ≥150 minutes per week and that its cardiometabolic benefits extend beyond weight loss per se, directly addressing systemic inflammation, endothelial dysfunction, atherogenic dyslipidemia, and hypercoagulability (19).

Lifestyle modification, such as caloric restriction, dietary quality improvement, aerobic and resistance exercise, sleep optimization, and stress reduction, constitutes the single most powerful intervention for preventing UCD initiation and reversing early-stage progression. A 5–10% weight loss achieves measurable reductions in VAT mass, hepatic lipid content, circulating ceramides, NF-κB activity, and TMAO levels, directly interrupting Stages I–IV of the continuum. Greater weight loss (≥10–15%) produces disease-modifying effects including remission of T2DM, resolution of MASLD, and regression of subclinical ASCVD (19). This evidence base positions primordial prevention, as a mechanistic imperative: preventing VAT dysfunction from arising is categorically more effective than targeting downstream UCD stages pharmacologically (14).

## 4 The unified cardiometabolic disease continuum

The UCD continuum can be conceptualized as an ordered, self-reinforcing mechanistic framework in which upstream metabolic disturbances generate molecular mediators that may promote, amplify, or accelerate downstream organ-specific phenotypes. This architecture should not be interpreted as a rigid linear sequence that occurs identically in all individuals. Rather, it represents a biologically plausible model integrating evidence from human imaging studies, epidemiological cohorts, translational physiology, biomarker studies, clinical guidelines, and major outcome trials. The strength of evidence varies across stages: some components, such as visceral adiposity, endothelial dysfunction, albuminuria, and cardiorenal amplification, are strongly supported by human clinical and epidemiological data, whereas others, including specific ceramide-, inflammasome-, microbiome-, and senescence-mediated pathways, remain partly dependent on translational or preclinical evidence.

Within this proposed framework, Stage I, visceral adipose tissue dysfunction, is positioned as the principal initiating event, supported most strongly by human imaging, epidemiological, and intervention data linking visceral adiposity to insulin resistance, MASLD, hypertension, ASCVD, HFpEF, and CKD risk. Stage II, portal free fatty acid flux and lipotoxicity, is proposed as a major biochemical consequence of dysfunctional VAT lipolysis and as a central bridge between adipose tissue dysfunction, hepatic steatosis, atherogenic dyslipidemia, skeletal muscle insulin resistance, and β-cell stress. Stages III, IV, and V, involving sterile inflammation, gut microbiome dysbiosis/TMAO signaling, and adipokine/β-cell dysregulation, are best interpreted as downstream or co-amplifying domains that may become activated once the upstream lipotoxic and inflammatory substrate is established.

Stage VI, endothelial dysfunction, represents a high-confidence vascular conversion point at which persistent metabolic, inflammatory, and oxidative stress is translated into clinically relevant vascular and microvascular injury. Stage VII, epicardial adipose tissue remodeling, is positioned as an organ-specific cardiac expression of systemic adipose and inflammatory dysfunction, with moderate evidence supporting its association with CAD, HFpEF, and atrial fibrillation. Stage VIII, impaired metabolic flexibility and mitochondrial dysfunction, is interpreted as an energetic convergence point integrating lipotoxic, inflammatory, endocrine, and physical inactivity-related stressors. Stage IX, the cardiorenal amplification loop, represents an advanced self-reinforcing phase in which established renal, vascular, and cardiac injury amplifies systemic inflammation, neurohormonal activation, endothelial dysfunction, and residual cardiometabolic risk.

**Figure 1:**
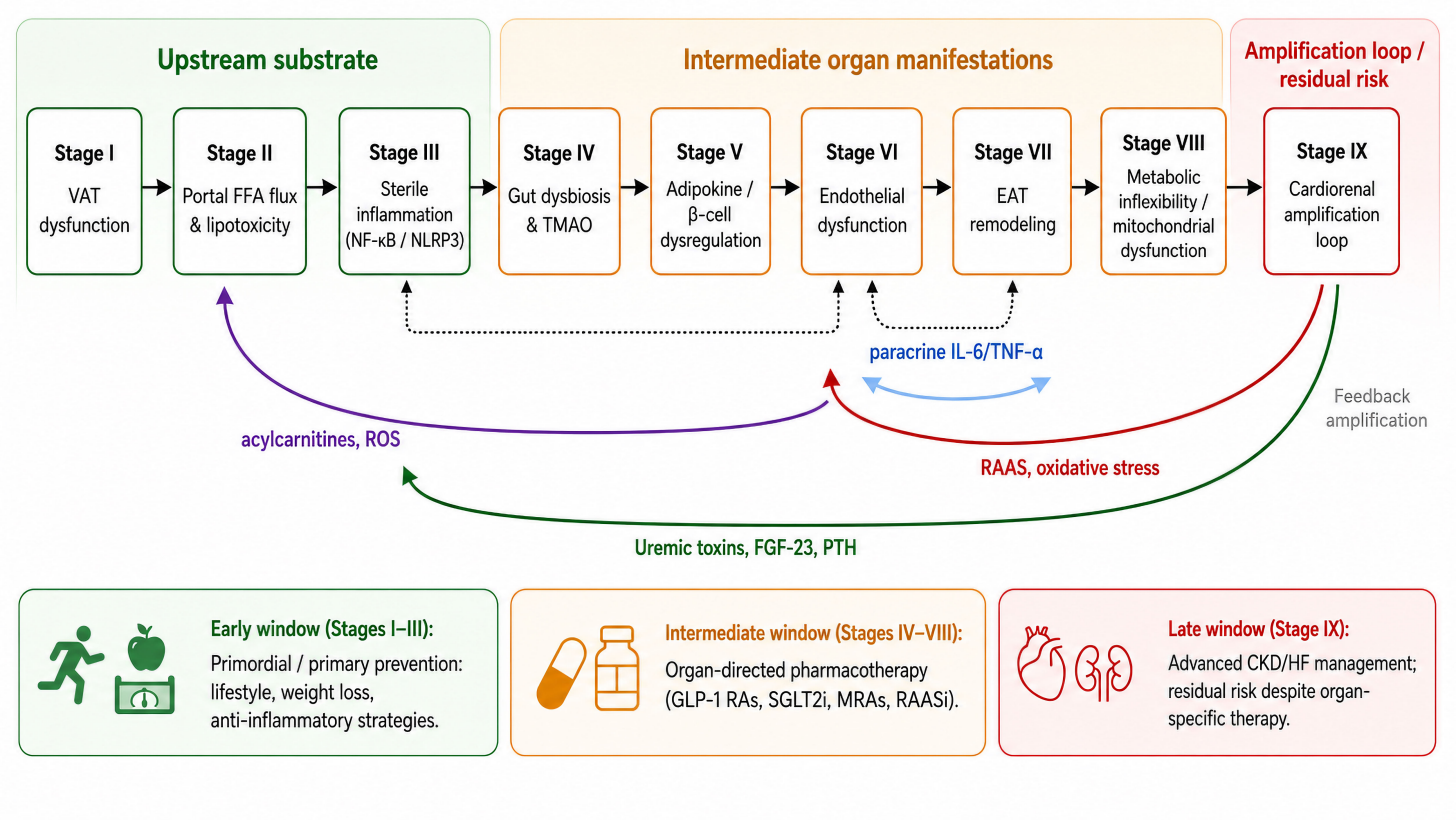
Causal architecture of the unified cardiometabolic disease (UCD) continuum. The nine UCD stages are shown as a primary causal chain from visceral adipose tissue (VAT) dysfunction through portal FFA flux, sterile inflammation, gut dysbiosis, adipokine/β-cell dysregulation, endothelial dysfunction, epicardial adipose tissue remodeling, and metabolic inflexibility/mitochondrial dysfunction to the cardiorenal amplification loop, with feedback arrows illustrating how acylcarnitines/ROS, RAAS activation, uremic toxins, FGF-23, and PTH propagate upstream stages. Color-banded “early”, “intermediate”, and “late” therapeutic windows highlight that primordial and primary prevention at Stages I–III yield the greatest potential to prevent or regress downstream cardiometabolic and cardiorenal manifestations.

This staged architecture has direct implications for intervention timing. Earlier interventions, particularly those that reduce visceral adiposity, improve metabolic flexibility, restore endothelial function, or interrupt renal and inflammatory amplification, are expected to produce broader downstream benefits than interventions applied after advanced organ damage has developed. However, the model should be interpreted as a mechanistic framework for hypothesis generation, biomarker development, and trial design rather than as proof that all individuals progress through a fixed, obligatory sequence. The 2026 AHA/ACC/ADA/ASN CKM staging construct is broadly compatible with this framework since it recognizes progressive cardiometabolic risk accumulation, although CKM remains primarily a clinical staging system rather than a fully mechanistic disease model (14). **Figure 1**.

### 4.1 Stage I: visceral adipose tissue dysfunction

White visceral adipose tissue (VAT), especially omental and mesenteric depots, is positioned in the proposed UCD framework as the principal upstream adipose substrate of cardiometabolic progression (20). Although VAT constitutes only a minority of total body fat, its proximity to the portal circulation and distinct metabolic activity confer a disproportionate association with insulin resistance, dyslipidemia, MASLD, hypertension, ASCVD, HFpEF, and CKD risk compared with subcutaneous adipose tissue (21). Under chronic positive energy balance, VAT adipocytes undergo progressive hypertrophy, local hypoxia, extracellular matrix remodeling, immune-cell recruitment, and impaired insulin-mediated suppression of lipolysis (8, 20). These processes generate a biological environment capable of promoting portal free fatty acid flux, ectopic lipid deposition, systemic inflammation, adipokine imbalance, endothelial dysfunction, and downstream organ injury.

Accordingly, VAT dysfunction should be interpreted as a high-confidence initiating domain within the UCD model, but not as an exclusive or universally obligatory entry point. Some individuals may express cardiometabolic risk through alternative or parallel pathways, including sedentary metabolic inflexibility, genetic susceptibility, early-life programming, ectopic fat despite normal BMI, pre-existing hypertension, or kidney dysfunction. Nevertheless, the VAT-as-initiator hypothesis remains strongly supported by human imaging, epidemiological, and intervention data showing that reduction of visceral adiposity is accompanied by improvement in multiple downstream cardiometabolic phenotypes.

Excess and dysfunctional adipose tissue serves as the primary upstream factor in the UCD continuum. This relationship is established as the initial stage in the pathophysiological progression of CKM, as well as in the development of diabetes, hypertension, hyperlipidemia, MASLD, inflammatory activation, and ultimately cardiovascular and kidney disease (14, 19). The UCD continuum can be initiated by the loss of the capacity to safely store caloric surplus in subcutaneous depots through healthy hyperplasia. The failure of VAT progenitor cells to sustain healthy hyperplastic expansion, attributable to elevated TGF-β signaling and accumulation of profibrotic progenitor subpopulations, promotes extracellular matrix remodeling and fibrosis (22). When subcutaneous adipose tissue expansion capacity is exceeded or functionally impaired, excess lipids are redirected toward ectopic depots in the liver, skeletal muscle, pancreas, and pericardium, driving organ-specific lipotoxicity independently of gross obesity (21). This mechanism could also explain the metabolically obese normal weight phenotype, in which individuals with a BMI below the conventional obesity threshold nevertheless harbor high VAT mass and substantial cardiometabolic risk.

The loss of functional brown and beige adipocyte activity, driven by impaired UCP-1 expression and ceramide-mediated suppression of thermogenic programming, further reduces energy dissipation and potentiates a positive energy balance that sustains VAT expansion (8). The VAT-as-initiator hypothesis is strongly supported by human imaging and epidemiological studies and by bariatric and lifestyle intervention data showing reversal of downstream phenotypes when VAT is selectively reduced (21, 23, 24).

### 4.2 Stage II: portal free fatty acid flux, lipotoxicity, and atherogenic dyslipidemia

Dysfunctional VAT exhibits heightened lipolysis and blunted insulin-mediated suppression, releasing excess non-esterified fatty acids (NEFAs) and glycerol into the portal circulation (20). Because portal venous delivery of fatty acids bypasses systemic buffering and directly loads the liver, it constitutes a uniquely concentrated lipotoxic stimulus on hepatocytes, the first mechanism by which Stage I VAT dysfunction produces the MASLD phenotype. This portal FFA flux drives hepatic *de novo* lipogenesis, very low-density lipoprotein overproduction, and accumulation of intrahepatic triglycerides (22). Hepatic lipid overload suppresses insulin receptor substrate-1 (IRS-1) signaling through protein kinase C-ϵ (PKCϵ) activation, establishing hepatic insulin resistance and unrestrained gluconeogenesis (20, 22).

Insulin resistance in this context is not confined to the liver but represents a pan-organ defect characterized by impaired phosphorylation of insulin receptor substrate proteins and downstream PI3K–Akt signaling, expressed in a stage-specific manner. In skeletal muscle, DAG and ceramides activate PKCθ and JNK-1, impairing GLUT-4 translocation and driving peripheral insulin resistance (21). Intrapancreatic fat via ceramide- and ER stress–dependent mechanisms further impairs β-cell secretory capacity, completing the hepatic–pancreatic lipotoxic stage of T2DM (22). *De novo* ceramide synthesis from portal palmitoyl−CoA via SPT, especially C16:0 species, activates PP2A, dephosphorylating and inactivating Akt/PKB, thereby reducing insulin−stimulated glucose uptake and promoting hepatocyte apoptosis and steatohepatitis (22, 25). The resulting dyslipidemic triad, hypertriglyceridemia, low HDL, and LDL, accelerates ASCVD phenotype (20). This shared ceramide–IRS–PI3K–Akt signature across liver, muscle, and β-cells illustrates why insulin resistance is not merely a risk factor but the common pathophysiologic medium in which MASLD, T2DM, and ASCVD co-develop (8, 10).

### 4.3 Stage III: chronic sterile inflammation

The chronic low-grade systemic inflammation that defines the UCD continuum is due to convergent activation of two innate-immune signaling modules: the canonical NF-κB pathway and the NLRP3 inflammasome (26, 27). Within dysfunctional VAT, saturated fatty acids, cholesterol crystals, and damage-associated molecular patterns (DAMPs) from necrotic adipocytes function as dual-signal activators: the LPS–TLR4–MyD88–NF-κB axis provides Signal 1 (priming), whereas ATP, cholesterol crystals, and urate crystals provide Signal 2 (NLRP3 oligomerization) (28). Activated NLRP3 drives caspase-1-dependent secretion of IL-1β and IL-18, which, along with TNF-α and IL-6 from M1-polarized macrophages, perpetuates a systemic inflammatory state, amplifying insulin resistance across all metabolically relevant organs, the mechanistic link that explains why the T2DM, ASCVD, and HFpEF phenotypes are so commonly co-expressed (29).

Osteoporosis and low bone mineral density intersect with this inflammatory environment through shared signaling pathways. TNF-α and IL-1β upregulate RANKL and promote osteoclastogenesis while suppressing Wnt/β-catenin–dependent osteoblast differentiation, shifting remodeling toward net bone resorption (30). Ceramide accumulation in osteoblasts (Stage II), RAAS overactivation with renal calcium loss (Stage VI), and adiponectin deficiency (Stage V) further impair osteoblast survival and differentiation, producing a high-turnover state with excessive resorption and inadequate formation that ties osteoporosis to the same inflammatory and lipotoxic substrate driving T2DM, ASCVD, and CKD within the UCD framework.

In myocardial tissue, sustained NLRP3 activity promotes cardiomyocyte hypertrophy, apoptosis, and interstitial fibrosis, directly contributing to the HFpEF phenotype through adverse cardiac remodeling and diastolic dysfunction (31). Senescent cells, which accumulate progressively in VAT, liver, and vascular endothelium during obesity and aging, secrete a senescence-associated secretory phenotype (SASP), a cocktail of IL-6, IL-8, PAI-1, matrix metalloproteinases, and pro-inflammatory cytokines, which perpetuates NF-κB and NLRP3 activation independently of nutrient excess, explaining the amplified UCD burden in older individuals, even in the absence of severe obesity.

### 4.4 Stage IV: gut microbiome dysbiosis, metabolic endotoxemia, and the TMAO axis.

The gut microbiome is an organ−level participant in UCD pathogenesis with both inflammatory and metabolic effects (32, 33) In dysbiosis, loss of SCFA−producing taxa (*Roseburia* spp., *Faecalibacterium prausnitzii*) and overgrowth of gram−negative bacteria downregulate tight−junction proteins (ZO−1, occludin, claudin−1), allowing LPS translocation into the circulation (32), Circulating LPS activates TLR4 and amplifies NF−κB signaling, linking gut ecology to the inflammatory amplifier that drives downstream UCD phenotypes, while SCFA depletion impairs GPR41/GPR43−mediated GLP−1 secretion and sustains M1 polarization and NF−κB–driven tissue inflammation (32).

Beyond LPS, the microbiome converts dietary choline, L-carnitine, phosphatidylcholine, and betaine into trimethylamine (TMA), which hepatic FMO3 oxidizes to trimethylamine N-oxide (TMAO) (33). TMAO promotes ASCVD by inhibiting hepatic CYP7A1, enhancing macrophage cholesterol influx, activating NLRP3, increasing thromboxane A2-mediated platelet reactivity, and suppressing the SIRT3–SOD2 axis (33). Elevated TMAO levels independently predict major adverse cardiovascular events (34). While depletion of cholesterol−catabolizing *Oscillibacter* spp. may further contribute to hypercholesterolemia, adding a lipid−modulating gut–heart link (33).

### 4.5 Stage V: adipokine dysregulation, GH/IGF-1 axis, and pancreatic β-cell failure

The transition from physiological to dysfunctional VAT is accompanied by alterations in adipokine secretion. Adiponectin, which exerts insulin-sensitizing, anti-inflammatory, and cardioprotective effects via AMPK activation and suppression of hepatic ceramide synthesis, is markedly reduced in proportion to VAT mass (29). The leptin-to-adiponectin ratio (LAR) has emerged as a clinically actionable composite biomarker of adipokine imbalance that correlates more strongly with insulin resistance and cardiovascular risk than adipokine alone. Beyond this, resistin elevates hepatic insulin resistance via TLR4 activation and is particularly enriched in EAT secretion (35); visfatin/NAMPT reinforces M1 macrophage polarization; and chemerin recruits innate immune cells into VAT, amplifying the Stage III inflammatory substrate (36).

Growth hormone resistance with reduced IGF-1 bioactivity adds a further endocrine layer to Stage V, in established obesity, hepatic GH receptor signaling is suppressed by elevated free fatty acids and IL-6, lowering hepatic IGF-1 synthesis, while hyperinsulinemia suppresses IGF-binding protein-1, altering IGF-1 bioavailability. At Stage V, IGF-1 normally supports pancreatic β-cell proliferation and survival via IGF-1R–IRS-2 signaling; functional IGF-1 deficiency therefore accelerates β-cell mass decline and hastens the transition from compensated insulin resistance to overt T2DM. At Stage VIII, concomitant IGF-1 deficiency reduces satellite cell activation and skeletal muscle protein synthesis, contributing to sarcopenia and thereby diminishing the principal site of insulin-stimulated glucose disposal and AMPK activation. Sarcopenic obesity thus represents a compound phenotype in which Stages I and VIII reinforce each other, increasing cardiometabolic risk beyond what BMI alone would suggest.

Pancreatic β-cell failure, which converts compensated insulin resistance into overt T2DM, is interpreted in the UCD framework as a downstream event promoted by prolonged exposure to lipotoxic, inflammatory, oxidative, and endocrine stress. Saturated FFAs can induce ER stress through the unfolded protein response, mitochondrial dysfunction with excess ROS generation, ceramide accumulation via SPT, and NLRP3-mediated caspase-1-dependent β-cell pyroptosis (28, 31). The interaction between β-cell ceramide accumulation and adiponectin deficiency may be particularly detrimental. Adiponectin normally activates ceramidase through AdipoR1/AdipoR2 receptors, degrading pro-apoptotic ceramide species; therefore, adiponectin deficiency may amplify ceramide-associated β-cell vulnerability (29). However, the timing and magnitude of β-cell failure vary substantially across individuals and are modified by genetic susceptibility, β-cell reserve, glucotoxicity, disease duration, and environmental exposures.

### 4.6 Stage VI: endothelial dysfunction, obesity-related hypertension, and cognitive impairment

Endothelial dysfunction can be viewed as a vascular threshold through which persistent metabolic dysregulation is translated into clinically relevant cardiovascular and microvascular disease. Under combined hyperglycemia, hyperlipidemia, and inflammatory cytokines, eNOS becomes uncoupled, shifting from nitric oxide to superoxide generation due to BH4 depletion (20). Reduced NO impairs vasodilation, increases leukocyte adhesion via ICAM−1, VCAM−1, and E-selectin, and accelerates atherosclerotic plaque progression, forming the ASCVD vascular substrate (21). Hyperglycemia-driven Advanced glycation end-products (AGEs) activate RAGE, stimulating NF-κB, VEGF, and HIF-1α, which further increases vascular permeability and sustains inflammation (20).

Endothelial dysfunction creates a prothrombotic vascular surface by increasing PAI−1, von Willebrand factor, and fibrinogen levels, generating a hypofibrinolytic state that markedly elevates acute arterial thrombosis risk, independent of plaque burden (27).

Cognitive impairment and vascular dementia represent neurological expressions of this same Stage VI, systemic mediators such as TMAO and pro-inflammatory cytokines from Stage III promote neuroinflammation and microvascular injury in the brain, while cerebrovascular endothelial dysfunction reduces autoregulatory capacity and renders hippocampal and cortical perfusion vulnerable to hypotension and microemboli (37). These processes position cognitive impairment not as an isolated complication of aging or diabetes but as a neurological endpoint of the UCD/CKM continuum.

Hypertension has a dual role in the UCD continuum, it is both a mechanistic consequence of upstream stages and an independent amplifier of downstream organ damage. In CKM, hypertension (SBP ≥130 mmHg or DBP ≥80 mmHg) is designated a Stage 2 criterion, reflecting its position as one of the earliest clinical manifestations of accumulated metabolic risk (14). First, overactivation of the renin–angiotensin–aldosterone system, driven by VAT-derived angiotensinogen and hyperinsulinemia, sustains vasoconstriction, sodium retention, oxidative stress, and vascular fibrosis, linking the UCD continuum to arterial hypertension and HFpEF (38, 39). Second, hyperinsulinemia consequent to Stage II insulin resistance activates renal tubular Na^+^/K^+^-ATPase, increasing distal tubular sodium reabsorption and expanding plasma volume. Third, sympathetic nervous system overactivation, driven by hypothalamic leptin resistance and elevated free fatty acid-mediated stimulation of the ventrolateral medulla, raises heart rate and peripheral vascular resistance.

Microvascular and microcirculatory dysfunction represent the small-vessel expression of Stage VI endothelial injury, affecting renal, myocardial, retinal, and cerebral beds through a shared substrate of eNOS uncoupling, oxidative stress, and pericyte loss. In the kidney it manifests as diabetic microangiopathy and albuminuria, in the heart as coronary microvascular dysfunction that underlies HFpEF (particularly in women), and in the retina as early diabetic retinopathy and retinal arteriolar narrowing (40), providing a unifying kidney–heart–retina–brain microvascular lesion within the UCD continuum.

At vascular level, eNOS uncoupling reduces nitric oxide–mediated vasodilation and renders resistance arterioles hyper-responsive to angiotensin II and catecholamines, perpetuating elevated systemic vascular resistance (20, 36). Perivascular adipose tissue (PVAT) surrounding resistance arteries undergoes the same pro-inflammatory phenotypic shift as VAT, releasing IL-6, TNF-α, and leptin into the adventitia and media of vessel walls, directly impairing smooth muscle relaxation independently of endothelial function. Coronary microvascular dysfunction due to endothelial NO deficiency and EAT-derived inflammation represents the dominant ischemic mechanism in women with HFpEF (39).

### 4.7 Stage VII: epicardial adipose tissue

Epicardial adipose tissue (EAT) exerts paracrine and vasocrine pathological effects directly on the heart (35). In the UCD context, EAT may represent an anatomical locus through which systemic metabolic dysregulation is translated into organ-specific cardiac pathology. EAT undergoes a phenotypic shift from thermogenic and cardioprotective to pro-inflammatory, profibrotic, and proatherogenic, releasing IL-6, TNF-α, resistin, and matrix metalloproteinases directly into the myocardium and coronary adventitia. An EAT thickness ≥7 mm (by echocardiography) or area ≥9 cm² (by computed tomography) is independently predictive of coronary artery disease, HFpEF, and atrial fibrillation (35).

Epicardial adipose tissue promotes HFpEF via TGF−β1/SMAD2/3 signaling, which drives collagen I/III deposition by cardiac fibroblasts and a titin shift from compliant N2BA to stiffer N2B, increasing myocardial passive stiffness and impairing diastolic filling, meanwhile EAT−derived IL−6 and TNF−α further suppress mitochondrial biogenesis and fatty−acid β−oxidation, creating the energetic inefficiency characteristic of HFpEF (39, 41). HFpEF thus emerges when the UCD continuum, initiated in VAT, remodels the heart through EAT, justifying its description as the “NASH of the heart,” paralleling MASLD’s hepatic lipotoxic fibrosis (5).

### 4.8 Stage VIII: impaired metabolic flexibility and mitochondrial dysfunction

Metabolic flexibility, the organism’s capacity to shift between glucose and fatty-acid oxidation in response to nutritional state, is an early and mechanistically central feature of the UCD continuum that may precede overt insulin resistance as a detectable phenotype (10, 18). In the UCD continuum, this flexibility is lost at the cellular level through ceramide- and DAG-mediated GLUT-4 sequestration in skeletal muscle, while the fasting transition to lipid oxidation is simultaneously impaired by mitochondrial dysfunction, generating incomplete fatty-acid oxidation products, long-chain acylcarnitines, that are independently cardiotoxic (18).

Physical inactivity and reduced mobility amplify these defects across multiple stages, sedentary behavior reduces skeletal muscle GLUT-4 expression and insulin-stimulated glucose uptake independently of obesity, worsening peripheral insulin resistance (Stage II). Inactivity lowers AMPK activation, which normally restrains hepatic gluconeogenesis, promotes mitochondrial biogenesis via PGC-1α, and maintains adiponectin production; AMPK hypoactivation therefore accelerates ceramide accumulation, reduces adiponectin, and increases the leptin-to-adiponectin ratio even without weight gain, mechanistically reproducing Stages II, V, and VIII in lean but inactive individuals (10, 18). The ADA recommend at least 150 minutes per week of moderate physical activity for individuals with diabetes or obesity, emphasizing benefits for glycemic control, cardiorespiratory fitness, and mortality that extend beyond weight loss (19). From the UCD perspective, this level of activity restores AMPK signaling, reduces VAT mass and ceramide flux, suppresses NF-κB and NLRP3, and improves mitochondrial β-oxidation efficiency (Stages I–IV and VIII), while resistance training further expands skeletal muscle GLUT-4 capacity and insulin-independent glucose disposal, lowering glycemic excursions that drive AGE formation and Stage VI endothelial injury.

Dysregulated coenzyme Q metabolism in hepatocytes drives excess superoxide via reverse electron transport at complex I, promoting hepatic insulin resistance through mitochondrial ROS−mediated IRS−1 serine phosphorylation, independently of ceramide and DAG (42). In cardiomyocytes, pathological reliance on fatty acid oxidation with reduced β-oxidation efficiency yields insufficient ATP for elevated filling pressures, causing mechanoenergetic uncoupling, a characteristic of HFpEF (41). Thus, impaired metabolic flexibility provides a shared energetic substrate for T2DM, HFpEF, and MASLD, reinforcing UCD unity.

### 4.9 Sex-specific modulation of the UCD continuum

The UCD continuum exhibits important sex-specific differences in molecular signaling and clinical expression. In premenopausal women, 17β−estradiol acting via ERα upregulates eNOS, suppresses RAAS, favors gluteofemoral over visceral fat distribution, and dampens NF-κB–mediated inflammation, slowing early continuum progression. Menopause-related estrogen loss accelerates visceral adiposity, dyslipidemia, and endothelial dysfunction, closing the premenopausal CVD risk gap within 5–10 years (43). EAT volume increases disproportionately in postmenopausal women, and its shift to a proinflammatory secretory profile is hormone-driven (35).

Reproductive dysfunction further reflects sex-specific expression of the same substrate: in women, polycystic ovary syndrome (PCOS) arises from VAT-driven hyperinsulinemia and hyperandrogenism (Stages I–II), is recognized as a CKM risk enhancer, and signals heightened future risk of T2DM and ASCVD, while adverse pregnancy outcomes (gestational diabetes, hypertensive disorders of pregnancy, preterm birth) similarly act as bidirectional CKM risk enhancers by both reflecting and accelerating progression along the UCD continuum. In men, VAT-associated aromatase activity and chronic inflammation promote functional hypogonadism, impairing spermatogenesis and positioning infertility as an organ-specific manifestation of visceral adiposity and Stage III inflammatory signaling rather than a primary gonadal disorder (43).

Clinically, HFpEF in women arises mainly from coronary microvascular dysfunction and diastolic impairment, whereas in men, it is more often linked to obstructive CAD and myocyte loss (44). Sex−chromosomal modulation of TLR4, NF−κB, and NLRP3 signaling creates a heightened inflammatory response in women, further amplified after menopause, while higher adiponectin at a given VAT mass partially buffers progression until estrogen withdrawal attenuates this protection (29).

### 4.10 The cardiorenal amplification loop

In the final escalatory stage of the UCD continuum, damage established in earlier modules is fed back and amplified through organ-to-organ signaling. The AHA’ CKM staging framework is most useful as a clinical tool for staging this advanced expression rather than as a mechanistic model (6). Uremic toxins, such as indoxyl sulfate and p−cresyl sulfate, derived from gut microbial metabolism and poorly cleared by diseased kidneys, cause endothelial injury, ROS production, and NF−κB activation, recapitulating systemic LPS-like endotoxemia (27). Elevated FGF−23 and PTH promote left−ventricular hypertrophy and fibrosis via FGFR4−mediated calcineurin–NFAT signaling and RAAS amplification (6). Concurrently, MASLD-related hepatic dysfunction drives hypoalbuminemia, dyslipidemia, and neurohormonal activation, creating a hepato−cardiac−renal loop that CKM’s liver omission does not fully capture (5).

Proteinuria, particularly albuminuria measured as urine albumin-to-creatinine ratio (UACR) ≥30 mg/g, is both a defining diagnostic criterion of CKD and an independent mediator of cardiovascular risk in the UCD continuum. The 2026 AHA/ACC/ADA/ASN CKM Guideline recommends annual dual assessment of eGFR and UACR in all individuals at CKM Stage 2 and above, reflecting the complementary and additive prognostic information these two markers provide regarding the risks of progressive CKD, cardiovascular events, and all-cause mortality (14). Mechanistically, albuminuria in the UCD context arises from multiple converging pathways: ceramide-mediated podocyte apoptosis, which erodes the glomerular filtration barrier; RAAS-driven glomerular hypertension, which elevates intraglomerular capillary pressure and enhances protein filtration; NLRP3-mediated tubular epithelial injury, which impairs tubular protein reabsorption; and endothelial glycocalyx degradation by circulating AGEs and reactive oxygen species, which increases glomerular permeability (45). Beyond its diagnostic utility, albuminuria actively amplifies the UCD continuum through triggering NF-κB-mediated interstitial inflammation and tubulointerstitial fibrosis, and as a marker of systemic endothelial dysfunction (Stage VI). Individuals with UACR ≥30 mg/g demonstrate impaired coronary, cerebrovascular, and peripheral microvascular reactivity, consistent with the pan-endothelial nature of Stage VI (46). SGLT2 inhibitors and RAASi reduce UACR through mechanisms that include renal hemodynamic improvement (reduction of intraglomerular pressure), NLRP3 suppression, and NF-κB inhibition, connecting albuminuria reduction directly to the mechanistic framework of the UCD continuum.

The cardiorenal loop explains the persistent residual risk, organ-specific therapies leave the upstream continuum intact, whereas mechanism-guided, multi-stage interventions, such as GLP-1 receptor agonists and SGLT2 inhibitors, are required to disrupt its self-reinforcing logic (3, 4).

### 4.11 Therapeutic implications

The UCD mechanistic framework argues against single-pathway interventions and favors agents that simultaneously engage multiple stages of the continuum. GLP-1 receptor agonists reduce visceral and epicardial adipose mass (Stage I, VII), lower hepatic lipid content (Stage II), attenuate systemic NF-κB signaling (Stage III), restore endothelial NO bioavailability (Stage VI), and reduce MACE, HFpEF events, and kidney outcomes (cardiorenal loop), precisely because all these phenotypes are stages of shared pathobiological continuum (4, 6, 35). SGLT2 inhibitors reduce intracardiac and renal NLRP3 activation (Stage III), restore mitochondrial substrate flexibility (Stage VIII), lower EAT volume (Stage VII), decrease intraglomerular pressure, and demonstrate efficacy in HFpEF in both diabetic and non-diabetic patients, supporting the interpretation that part of their benefit is mediated through mechanisms extending beyond glucose lowering (27, 41, 44). Finerenone, a non-steroidal mineralocorticoid receptor antagonist, directly targets RAAS overactivation, bridging Stage VI endothelial dysfunction to the cardiorenal loop, independently reducing both cardiac fibrosis and CKD progression (4).

**Figure 2:**
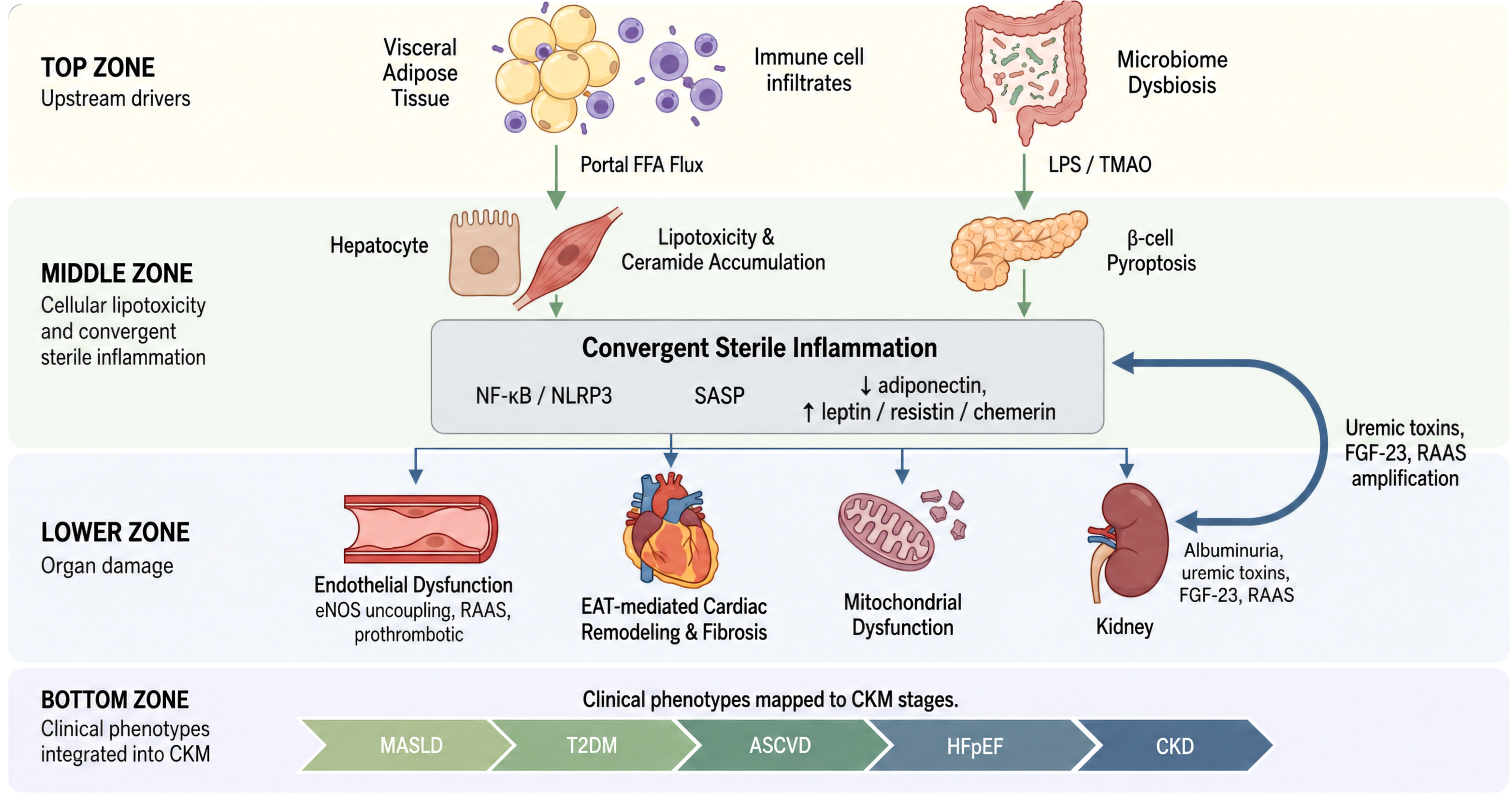
Schematic representation of the unified cardiometabolic disease (UCD) continuum. Upstream drivers in visceral adipose tissue and the gut microbiome (top zone) induce portal FFA flux, lipotoxic ceramide accumulation, β-cell pyroptosis, and microbiome-derived LPS/TMAO. These insults converge into NF-κB/NLRP3-mediated sterile inflammation with SASP-driven senescence and adipokine dysregulation (middle zone), leading to endothelial dysfunction, epicardial adipose tissue–mediated cardiac remodeling, mitochondrial impairment, and kidney injury (lower zone). The resulting cardiorenal amplification loop sustains progression toward clinically overt T2DM, MASLD, ASCVD, HFpEF, and CKD, which are eventually captured within the AHA cardiovascular–kidney–metabolic (CKM) framework. Upstream susceptibility factors, including genetic/epigenetic programming, HPA-axis dysregulation, impaired appetite regulation, sedentary behavior, dietary sodium excess, and adverse social/environmental exposures, modulate the threshold at which this continuum is initiated.

In addition to GLP−1 RAs, SGLT2 inhibitors, and finerenone, emerging strategies are increasingly targeting specific UCD stages. Dual or triple incretin agonists (GLP−1/GIP ± glucagon) aim to reduce VAT (Stage I). Bempedoic acid and PCSK9 inhibitors address atherogenic dyslipidemia (Stage II). Selective NLRP3 inhibitors and IL−1β antagonists (colchicine and canakinumab) target sterile inflammation (Stage III). FMO3−targeting strategies and Mediterranean dietary patterns modulate the TMAO axis (Stage IV). SPT inhibitors and other ceramide-lowering approaches target lipotoxicity (Stages II–V), whereas senolytics address SASP-driven inflammation (Stage III). Crucially, GLP−1 RAs, SGLT2 inhibitors, and finerenone already have Level I evidence from large cardiovascular outcome trials, whereas selective NLRP3 inhibitors, SPT inhibitors, and senolytics remain at the preclinical or early−phase stages and should not yet be considered on equal therapeutic footing (**Figure 2**; **Supplementary Table 2**).

## 5 Discussion

T2DM, ASCVD, HFpEF, MASLD, HT, and CKD are phenotypic endpoints of a unified pathophysiological process. This does not negate their distinct clinical and coding identities but reframes them as organ-dominant stages in the same underlying continuum. This continuum is driven by recurrent mechanistic axes, visceral adiposity and ceramide-mediated lipotoxicity, NF−κB/NLRP3−dependent sterile inflammation, gut microbiome–derived endotoxemia and TMAO, adipokine dysregulation, endothelial dysfunction, impaired mitochondrial metabolic flexibility, and cardiorenal amplification, whose organ-specific dominance determines whether the clinical threshold is first crossed in the pancreas, coronary artery, left ventricle, liver, or kidney. Within this network, ceramide metabolism is the best-defined lipotoxic pathway and a high-priority therapeutic target, whereas the TMAO axis provides a key gut–vascular link. The pleiotropic benefits of GLP−1 receptor agonists, SGLT2 inhibitors, and finerenone across glycemic, cardiovascular, renal, and hepatic endpoints strongly support the UCD thesis: their effects are most coherently viewed as modulation of the continuum rather than coincidental multi−organ actions. Importantly, the UCD model should be interpreted as a mechanistic and integrative framework rather than as evidence that every individual progresses through a fixed, obligatory, or strictly linear trajectory. Instead, the proposed continuum reflects convergent biological axes that may emerge in different orders, intensities, and organ-dominant patterns depending on genetic background, sex, adipose tissue distribution, environmental exposure, lifestyle, comorbidities, and disease duration.

### 5.1 Limitations

This narrative integrative review was susceptible to selection bias. Much of the mechanistic data for ceramide, NLRP3, and gut microbiome axes derives from preclinical models, and translation to human pathophysiology is not always direct or quantitatively established. Sex-specific data remain underrepresented in many mechanistic studies, and UCD pathophysiology in non-binary populations and across the pregnancy continuum was not addressed. The therapeutic landscape is evolving rapidly (triple incretin agonists, oral GLP-1 RAs, NLRP3 inhibitors), and any therapeutic staging should be interpreted as a current snapshot rather than a definitive guide.
