# Supplementary material for: The unified cardiometabolic disease continuum: mechanistic stages of a single pathophysiological process
Source: Front Endocrinol (Lausanne). 2026 Jul 9;17:1897556. doi: 10.3389/fendo.2026.1897556 (PMC13391316; doi:10.3389/fendo.2026.1897556)
Supplement: Supplementary file 1 [file Table1.docx]

**Supplementary Table 1. Strength and reliability of evidence supporting the proposed unified cardiometabolic disease continuum**

| **UCD stage / mechanistic domain** | **Core claim in the UCD model** | **Main type of supporting evidence** | **Reliability of evidence** | **Interpretation within the UCD framework** | **Main limitation / caution** |
| --- | --- | --- | --- | --- | --- |
| Etiological drivers | Genetic susceptibility, early-life programming, chronic stress, circadian disruption, sedentary behavior, and adverse social environments increase susceptibility to pathological visceral adiposity and cardiometabolic progression. | Human genetic studies, epigenetic studies, epidemiological cohorts, mechanistic endocrine studies, and guideline-level recognition of social determinants and lifestyle risk. | **Moderate to high** | These factors are best interpreted as susceptibility and amplification drivers rather than direct stages of the continuum. They determine the threshold at which visceral adipose tissue dysfunction becomes clinically relevant. | The relative contribution of each upstream driver varies across populations, sex, ancestry, socioeconomic context, and life-course exposure. |
| Stage I: Visceral adipose tissue dysfunction | Dysfunctional visceral adipose tissue is positioned as the principal initiating biological substrate of the UCD continuum. | Human imaging studies, epidemiological cohorts, adipose tissue biology studies, intervention studies showing metabolic improvement after visceral fat reduction, and guideline-level recognition of adiposity as an early CKM driver. | **High** | This is one of the strongest components of the model. VAT dysfunction provides a biologically coherent starting point linking obesity, insulin resistance, MASLD, dyslipidemia, hypertension, ASCVD, HFpEF, and CKD risk. | VAT is not the only possible entry point. Some individuals may enter the continuum through genetic risk, sedentary metabolic inflexibility, ectopic fat despite normal BMI, or pre-existing hypertension/CKD. |
| Stage II: Portal free fatty acid flux, ectopic lipid deposition, ceramide-mediated lipotoxicity, and atherogenic dyslipidemia | Increased VAT lipolysis promotes portal FFA delivery, hepatic lipid accumulation, insulin resistance, ceramide generation, and atherogenic dyslipidemia. | Human metabolic studies, liver and adipose tissue studies, translational mechanistic studies, lipidomics, and consistent associations with MASLD, T2DM, and ASCVD phenotypes. | **Moderate to high** | The portal FFA/lipotoxicity axis is a central biochemical bridge between VAT dysfunction and organ-specific metabolic disease. | The role of specific ceramide species is biologically plausible and increasingly supported, but causal thresholds and clinical cutoffs are not yet standardized for routine risk staging. |
| Stage III: Chronic sterile inflammation via NF-κB and NLRP3 inflammasome activation | VAT dysfunction, lipotoxicity, DAMPs, cholesterol crystals, LPS, and metabolic stress activate NF-κB/NLRP3 signaling, generating systemic low-grade inflammation that amplifies insulin resistance, vascular injury, myocardial remodeling, and kidney damage. | Translational studies, human biomarker studies, preclinical disease models, inflammatory pathway studies, and clinical trial evidence for selected anti-inflammatory interventions in cardiovascular disease. | **Moderate** | This stage is a credible inflammatory amplifier connecting metabolic dysfunction with cardiovascular and renal injury. | A substantial portion of NLRP3-specific mechanistic evidence remains preclinical or translational. Human causal evidence is stronger for systemic inflammation than for each specific inflammasome-mediated link. |
| Stage IV: Gut microbiome dysbiosis, metabolic endotoxemia, and TMAO axis | Dysbiosis, increased intestinal permeability, LPS translocation, reduced SCFA production, and increased TMAO generation contribute to inflammation, insulin resistance, endothelial dysfunction, thrombosis, and ASCVD risk. | Human observational cohorts, metabolomic studies, mechanistic microbiome studies, animal models, and meta-analyses for TMAO-associated cardiovascular risk. | **Moderate for association; low to moderate for intervention** | The gut–metabolic–vascular axis is a plausible and clinically relevant amplifier of the UCD continuum. | Associations between TMAO and outcomes are stronger than evidence that directly modifying TMAO or microbiome composition improves hard cardiometabolic endpoints. Diet, renal function, medication exposure, and host genetics confound interpretation. |
| Stage V: Adipokine dysregulation, GH/IGF-1 axis alteration, and pancreatic β-cell failure | Reduced adiponectin, increased leptin/adiponectin ratio, resistin, chemerin, visfatin/NAMPT, altered GH/IGF-1 signaling, and β-cell lipotoxicity contribute to the transition from compensated insulin resistance to overt T2DM and systemic cardiometabolic progression. | Human endocrine and adipokine studies, β-cell biology, translational models, and clinical associations with insulin resistance, T2DM, and cardiovascular risk. | **Moderate** | This stage explains how dysfunctional adipose tissue becomes an endocrine organ driving β-cell stress, insulin resistance, and systemic metabolic deterioration. | Many adipokines are context-dependent and not yet standardized as clinical biomarkers. The GH/IGF-1 axis is biologically relevant but less established as a routine staging component of cardiometabolic disease. |
| Stage VI: Endothelial dysfunction, microvascular injury, hypertension, and cognitive impairment | Persistent hyperglycemia, dyslipidemia, inflammation, oxidative stress, RAAS activation, and adipokine imbalance impair endothelial function and convert metabolic dysfunction into vascular, renal, cardiac, retinal, and cerebral injury. | Strong human vascular biology, epidemiological studies, clinical cohorts, hypertension and CKM guidelines, microvascular disease studies, and intervention evidence targeting blood pressure, lipids, glucose, RAAS, and kidney risk. | **High** | This is a high-confidence conversion point where upstream metabolic disease becomes clinically measurable organ damage. | Endothelial dysfunction is a convergent pathway rather than a single disease-specific mechanism. Cognitive impairment links are plausible but more heterogeneous and multifactorial than ASCVD, CKD, or hypertension links. |
| Stage VII: Epicardial adipose tissue remodeling | EAT shifts from a cardioprotective depot to a pro-inflammatory, profibrotic, and proatherogenic tissue that contributes to coronary artery disease, atrial fibrillation, and HFpEF. | Human imaging studies, echocardiography/CT-based studies, clinical associations with CAD/HFpEF/AF, and translational mechanistic studies. | **Moderate** | EAT provides an anatomically coherent bridge between systemic metabolic dysfunction and organ-specific cardiac remodeling. | Evidence is strong for association and biological plausibility, but less definitive for EAT as an independent causal driver rather than a marker of global visceral adiposity. |
| Stage VIII: Impaired metabolic flexibility and mitochondrial dysfunction | Loss of substrate switching, impaired AMPK signaling, mitochondrial inefficiency, incomplete fatty acid oxidation, and acylcarnitine accumulation contribute to insulin resistance, MASLD, skeletal muscle dysfunction, and HFpEF energetics. | Human metabolic physiology, exercise studies, mitochondrial biology, translational studies, and clinical evidence showing cardiometabolic benefit of physical activity independent of weight loss. | **Moderate** | This stage explains why cardiometabolic risk can progress even before overt obesity and why exercise has disease-modifying effects across the continuum. | Measurement of metabolic flexibility is not standardized in clinical practice, and mitochondrial dysfunction is difficult to assign temporally within the continuum. |
| Stage IX: Cardiorenal amplification loop | CKD, albuminuria, RAAS activation, uremic toxins, FGF-23/PTH excess, endothelial injury, and cardiac remodeling create a self-reinforcing heart–kidney–metabolic loop. | Human cohorts, CKD/CVD epidemiology, albuminuria risk prediction studies, CKM guideline statements, and large clinical trials of SGLT2 inhibitors, RAAS blockade, and finerenone. | **High** | This is one of the most clinically validated portions of the UCD model and explains persistent residual risk once organ damage is established. | The loop is well supported clinically, but it represents an advanced amplification phase rather than proof that all patients follow the same linear sequence from Stage I to Stage IX. |
| Therapeutic convergence across GLP-1 receptor agonists, SGLT2 inhibitors, finerenone, lipid-lowering therapy, and lifestyle intervention | Therapies that act across metabolic, cardiovascular, renal, hepatic, inflammatory, and adiposity-related pathways support the concept that these diseases share common biological substrates. | Large cardiovascular and renal outcome trials, diabetes/obesity/CKM guidelines, meta-analyses, and mechanistic substudies. | **High for GLP-1 receptor agonists, SGLT2 inhibitors, finerenone, lifestyle, RAAS inhibition, and lipid-lowering therapy; low to moderate for emerging therapies** | Therapeutic convergence provides strong pragmatic support for the UCD framework because several interventions improve outcomes across multiple organ systems. | Cross-organ therapeutic benefit supports shared biology but does not by itself prove a single obligatory causal continuum. Emerging approaches such as selective NLRP3 inhibitors, SPT inhibitors, microbiome-targeted therapies, and senolytics remain investigational. |

**Evidence reliability categories: High reliability** indicates support from consistent human data, major clinical guidelines, large outcome trials, meta-analyses, or reproducible human cohort/imaging evidence. **Moderate reliability** indicates support from consistent human observational or translational evidence, with strong biological plausibility but incomplete causal or interventional confirmation. **Low or emerging reliability** indicates evidence derived mainly from preclinical studies, early-phase trials, biomarker associations, or mechanistic plausibility without definitive human outcome validation.
