# Supplementary material for: The unified cardiometabolic disease continuum: mechanistic stages of a single pathophysiological process
Source: Front Endocrinol (Lausanne). 2026 Jul 9;17:1897556. doi: 10.3389/fendo.2026.1897556 (PMC13391316; doi:10.3389/fendo.2026.1897556)
Supplement: Supplementary file 2 [file Table2.docx]

**Supplementary Table 2. Mechanistic Stages of the Unified Cardiometabolic Disease Continuum: Biomarkers and Therapeutic Targets**

| **Stage** | **Mechanistic Axis** | **Key Molecular Mediators** | **Clinical Biomarkers / Thresholds** | **Therapeutic Targets** |
| --- | --- | --- | --- | --- |
| I | VAT dysfunction | HIF-1α, MCP-1, TGF-β, M1 macrophages, crown-like structures | Waist circumference, visceral adiposity index, CT/MRI VAT volume | GLP-1 RAs, bariatric surgery, exercise, caloric restriction |
| II | Ectopic lipid & lipotoxicity | NEFAs, DAG, ceramides (C16:0), PKCε/θ, PP2A, IRS-1 | Plasma ceramide score (CERT2), hepatic fat fraction (MRI-PDFF), IMCL | SPT inhibitors, pioglitazone, metformin, dietary lipid modulation |
| III | NF-κB/NLRP3 sterile inflammation | IL-1β, IL-18, TNF-α, IL-6, ASC speck, caspase-1, SASP | hs-CRP, IL-6, IL-1β, soluble TNF receptors | Colchicine, canakinumab, selective NLRP3 inhibitors, senolytics |
| IV | Gut microbiome & TMAO | LPS, TLR4, TMA/FMO3/TMAO, SCFA depletion | Plasma LBP, zonulin, TMAO (pooled RR ≈1.94 for MACE) | Mediterranean diet, fiber/prebiotics, FMO3 inhibitors, probiotics |
| V | Adipokine dysregulation & β-cell failure | ↓ Adiponectin, ↑ leptin, resistin, chemerin, visfatin; ER stress; β-cell pyroptosis | Leptin/adiponectin ratio (LAR), HOMA-IR, fasting C-peptide | GLP-1 RAs, dual incretin agonists, adiponectin mimetics |
| VI | Endothelial dysfunction | eNOS uncoupling, BH4 depletion, ICAM-1, VCAM-1, RAAS, PAI-1, AGE/RAGE | Flow-mediated dilation, PAI-1, vWF, ADMA, coronary flow reserve | ACEi/ARBs, finerenone, statins, BH4 supplementation, SGLT2i |
| VII | Epicardial adipose tissue (EAT) | IL-6, TNF-α, resistin, MMPs, TGF-β1/SMAD2/3, titin N2B shift | EAT thickness ≥7 mm (echo) or ≥9 cm² (CT), fat attenuation index | SGLT2i, GLP-1 RAs, ablation-adjunctive therapy |
| VIII | Metabolic flexibility & mitochondria | Long-chain acylcarnitines, coenzyme Q dysregulation, RET, ROS | Respiratory quotient response, acylcarnitine panel, hyperpolarized ¹³C MRS | Exercise training, SGLT2i, ketogenic strategies, mitochondrial-targeted antioxidants |
| IV. Modifier | Sex-specific axis | ERα / eNOS / NF-κB suppression; postmenopausal RAAS upregulation | Sex-stratified risk scores, coronary microvascular reserve | Sex-specific risk stratification; menopausal hormone therapy |
| X. Final | Cardiorenal amplification loop | Indoxyl sulfate, p-cresyl sulfate, FGF-23, PTH, RAAS, uremic NF-κB | eGFR, UACR, FGF-23, CKM stages 0–4 | Finerenone, SGLT2i, GLP-1 RAs, ACEi/ARBs, integrated CKM-guided care |
